# Practical Approaches for Size Estimation of High-Risk Groups in HIV/AIDS Control Program in Iran

**Published:** 2019-02

**Authors:** Salman KHAZAEI, Ensiyeh JENABI, Shahab REZAEIAN

**Affiliations:** 1. Research Center for Health Sciences, Hamadan University of Medical Sciences, Hamadan, Iran; 2. Pediatric Developmental Disorders Research Center, Hamadan University of Medical Sciences, Hamadan, Iran; 3. Research Center for Environmental Determinants of Health, Kermanshah University of Medical Sciences, Kermanshah, Iran

## Dear Editor-in-Chief

Acquired Immune Deficiency Syndrome (AIDS) is an emerging disease which has become known as the plague of the century. The estimated number of infected people with HIV in Iran is 71000 (53000–100000) in 2012 (1). The prevalence in the overall population is below 1%; this rate, however, has surpassed 5% in some high-risk groups such as injecting drug users (IDUs) (2). The country’s HIV epidemic is concentrated among IDUs, 68.1% of cases are due to needle-sharing versus 12.7% to sexual transmission. The highest trend of HIV was estimated to be a shift from IDUs to female sex workers (FSWs), and newly infected cases via unsafe sex will increase in the future (1).

Estimating the population size of hard to reach subgroups is an important entity in public health, especially in the HIV/AIDS control program (3). Having reliable estimates of the size of these groups (especially FSWs and IDUs in our country) is necessary for better tracking the epidemic, program planning, advocacy and enough resource allocations (3). This information is more need for countries like Iran with a concentrated HIV epidemic among IDUs and with limited resources (2).

There are few published statistics is available about population size of these groups using a standard and validated methodology in Iran (4–6). In order to improve the current surveillance system, it is crucial to having acceptable population size of these groups. However, such groups are hidden in the community, mainly because of strong stigmatization and legal punishment. Therefore they have reluctant to be identified as such and the number of them is prone to underestimation (7).

Special techniques have been introduced for size estimation of these groups including capture-recapture (CRC), multiplier (MP), and Networks Scale Up (NSU) techniques (8). Direct CRC and MP methods need direct contact between research team and high-risk groups. These techniques are appropriate to estimate sizes in a close population. However, for planning, we need to have national estimates. Indirect CRC and NSU which needs no direct contact with individuals with high-risk groups (8).

Some challenges like stigma and discrimination against such population in Iran with an Islamic culture have caused the direct estimation techniques to approximate their population size is inappropriate, also because of their small population size in different societies, any survey for direct estimation requires a very large sample size, which is also not often feasible. Although the indirect size estimation methods are commonly used in our country for these groups (9), the underestimate rate of the population still remains considerable in Iran.
